# 

**Published:** 1872-01-01

**Authors:** 


### CONTENTS OF VOLUME XIII.

### A.

PAGE.
Andrews, E. Clinical Cases in Surgery ... 6
Antiseptic Treatment in Injury of Elbow ... 6
Alumni Association of Jefferson Medical College ... 10
Ashurst, John, jr., on Principles and Practice of Surgery ... 10
American Ophthalmological Society, Transactions of ... 11
Albuminoid Disease of the Kidneys, Treatment of ... 14
Aphasia. By J. B. Lyman, M.D. ... 17
American Journal of Obstetrics, etc. ... 25
Anstie, Francis E., on Neuralgia and Diseases Resembling it 27
American Practitioner, a Monthly Journal ... 27
Asthma, Hydrate of Chloral in Treatment of ... 34, 256
Alcohol, Declaration of Medical Opinion in Extract ... 42, 77, 41
Astragalus and Os Calcis, Complete Excision of ... 47
American Medical Association, Notice ... 47, 74, 90, 105, 137, 153, 157, 286, 327, 343, 352, 362, 378
Alumni Meeting of Clicago Medical College ... 58, 73, 94
Abortion, Criminal. By E. Van De Warker, M. D. ... 63
Alcohol, Injection of into Cysts ... 63, 317
Amenorrhœa ... 72, 242
Acnæ ... 72
Aqua Ammonia, Inhalation for Whooping Cough ... 72
Apology ... 73
Appointments ... 94
Association of American Medical Editors ... 105, 138, 139
Anæsthesia ... 112
Acid, Carbolic, as Local Anæsthetic ... 140, 143, 349, 112
Aural Catarrh, Lectures on ... 144
Arterial Compression in Muscular Spasms ... 144
Aneurism of Popliteal Artery ... 156
American Association for Advancement of Science ... 204, 288
Aneurism, Surgical Treatment of ... 205
Aconite, Crystallized, Physiological Action of ... 228
Anti-Phlogistics in Myelitis ... 256
Alcohol and Opium, Effects of ... 259
Anasarca, Surgical Treatment of ... 261
Association, British Medical ... 266, 313
Anæmia, Progressive Pernicious ... 269
American Text-Books and Teachers ... 269
Ammonia, Hydrochlorate of ... 283, 284, 301
Aphonia, Cure ... 283
Aconite in Acute Pneumonia ... 283
Aspiration in Strangulated Hernia ... 286
Albuminuria and Cirrhosis in Same Patient ... 292, 300
Adolescents, Surgical Affections of ... 349
Ankle, Caries of ... 227
Artificial Placental Respiration ... 369

### B.

Baths, for Penis. By Prof. E. H. Andrews ... 7
Bromide Calcium, and Iron, and Potassium ... 14, 63, 208
Bliss & Torrey, Surgical Instruments ... 25
Barth, M., elected President ... 48
Bright's Disease, with Partial Hemiplegia ... 69, 93
Bronchitis, Rheumatic ... 71
Beef, Extract ... 109
Bonsor, Otho, M.D., Death of ... 110
Bed-Sores, Nitrate Silver in ... 141, 350
Bruit de Diable ... 143, 167
Bowel-Affections of Children ... 182, 234
Bromo-Chloralum ... 202, 207
Blindness and Deafness after Cerebro-Spinal Meningitis ... 251
Bladder, Rupture of ... 268
Blisters in Pneumonia ... 304
Bones, Diseases of ... 304, 239
Bowel Lesions of Typhoid Fever ... 142
Bioplasm. Address by Prof. Nelson ... 305
Bullock's Blood, in Phthisis, etc. ... 351
Brown-Sequard—Resigned ... 351
Beriberi, Case of ... 361
Bad Air, Effects of ... 361

### C.

Catarrh ... 324
Calculus, Mulberry and Phosphate ... 6
PAGE.
Cancer. By J. W. Bright, M.D. ... 11, 31, 121, 177
Commissioners of Public Charities, State of Illinois ... 11
Chicago Medical Society, Proceedings of ... 11, 27, 107, 280, 295
Cholera ... 15, 28, 153, 206
Chicago Medical College ... 25, 44, 73, 261
Consumption, Pulmonary ... 27, 67, 124, 141, 177, 273
Clavicle, Fracture of ... 40
Chicago Relief and Aid Society ... 45
Cystitis in Female ... 91
Cerebro-Spinal Meningitis ... 97, 117, 121, 132, 134, 211
Correspondence, Foreign, etc. ... 106, 149, 214
Conium in Acute Mania ... 120
Cundurango ... 129, 261
Cancrum Oris ... 133
Croup, Lime Inhalation in ... 135, 140, 184
Catheter, its Employment in Old People ... 136
Chicago College of Pharmacy ... 153, 233
Calculus Disease, Early Treatment of ... 170
Chicago Society of Physicians and Surgeons ... 176
Climate for Cancer and Consumptives ... 177
Cantharides. By Squibb ... 187
Carcinoma Calli, Diagnosis of ... 205
Clinton County Medical Society ... 217
Clinical Cases and Incidents ... 230, 257, 292, 371, 339, 355, 371
Chlorosis ... 241

### D.

Death, Presentiment of ... 7
Diphtheria ... 14
Dysentery, Chlorate Potassium in ... 32, 96
Diarrhœa, Chronic ... 39
Delirium Tremens, Chloral in ... 65
Diabetes ... 96, 206
Doctors in Russia ... 176
Drains, Smells and Fevers ... 192

### E.

Eye, Injuries of, etc. ... 4
Elbow, Compound Dislocation of, etc. ... 6, 7
Erysipelas ... 39
Exhibition, Medical ... 42, 365
Epilepsy ... 77, 155
Earth, Dry, in Surgery ... 80
Expiration, Prolonged ... 109
Emphysema, Treatment of ... 142
Epulis, Malignant, of Lower Jaw ... 169
Enuresis ... 171, 191
Explanation ... 185
Epistaxis, New Treatment of ... 221
Exsection of Shoulder ... 226
Empyemia, Antiseptic Injections in ... 370

### F.

Fraternal ... 9
Forceps, Application of ... 24
Funis Umbilicalis, Disease of ... 33
Female Physicians ... 53
Fungus as a Cause of Whooping Cough ... 62
Fractures ... 64
Food, Soluble, Liebig's, in Children ... 278

### G.

Gastritis ... 276
Gleanings from Exchanges ... 254, 255, 256, 260, 269, 281, 297, 331, 345, 366, 376
Galvanism, Therapeutic Value of ... 236
Granulations of Conjunctiva ... 168
Goitre ... 72
Geneva Medical College ... 74
Glycerine, in Hypodermic Injections ... 141
Gangrene, Hospital, Camphor in ... 152, 228

**H.**

PAGE.

Hydrophobia 212
Hysteria, its Pathology 193
Hemoptysis and Phthisis 154
Hydrocele 136
Hernia, Strangulated 136, 218
Herniopuncture 136
Hæmorrhoids in Pregnancy 103
Human Physiology 26
Heart, Valvular Disease of 37
Hydrocephalus, Infantile 40
Head, Injury of 49, 50
Hips, Dislocation of 49
Health, State Boards of 78
Hip-Joint, Excision of 87
Headaches, Periodical 93, 240

**I.**

Intemperance in France 43, 77
Indian Doctors, Fate of 64
Intussusception 68, 184
Illinois State Medical Society 105, 153, 169, 171
Insanity, its Treatment 112
Inhalation of Lime in Croup 135
Iodine in Vomiting 140
Indiana State Medical Society 154
Inoculation of Gray Tubercle 156
Illinois Medical Society, Central 203
Illinois Institution for Feeble-minded Children 204
Instruction, Clinical 217
Injections, Hypodermic 220, 221
Inebriate Asylum, N. Y. 222
Ingrowing Toe-nail 237
Intestinal Erectile Tumors 238
Intestinal Irritation 354
Iris, Tumor of, Removed 369

**K.**

Kidney, Albuminoid Disease of 14
Kidney, Removal of During Life 30
Kidney, Destroyed by Suppuration, etc. 289
Kidney, Encephaloid Disease of 326

**L.**

Lithotomy 65, 226, 263
Lung Test 171, 224
Library of Surgeon-General's Office 224
Luxation of Humerus 242
Lecture, Clinical, in Mercy Hospital 243
Laryngitis, Chronic 337

**M.**

Medical Directory and Register for Chicago 233
Military Tract Medical Association 185
Maxilla, Inferior, Removal of 169
Medical Science, its Origin 145, 161
Medical Societies 122
Marriage and Celibacy 46
Malformations 7
Medical Examiner 9
Minnesota State Medical Society 10, 61
Medical Faculty of Vienna 11
Medical Colleges and Graduates 15
Mortality of Chicago 15, 32
Money Receipts 16, 32, 48, 64, 96, 112, 128, 160, 208, 256, 320, 368
Medical Profession, its Needs, etc. 85
Medical Instruction in Summer 90
Menses, Suppression of 102
Milk as Diet in Lactation 219

**N.**

Neuralgia and Diseases Resembling it 27
Nitrous Oxide Liquid as an Anæsthetic 34
Necrologist's Report 94, 111
New York State Medical Society 112
Narceine, Hydrochlorate of 184
Nursing Sore Mouth 312

**O.**

Opium Cure 6
Ovariotomy 13, 64, 92
Omaha Medical Society 62
Ophthalmia, Granular. By Quinimus 77
Omentum, Carcinoma of 199
Ozone Tests 207

**P.**

PAGE.

Pruritus Vulva 241
Prize Essays 9, 365
Pathological Histology, Hand-Book 10
Pathology and Morbid Anatomy, Introduction to 10
Pregnancy, Phosphates in 14
Physicians' Relief Committee, Reports 25, 89
Pneumonia, Treatment of 30
Pleurisy, with Effusions 71, 252, 253
Putty, Physiological Action of 77
Pessary, New 91
Pepsin, its Digestive Power 109
Parasites, Animal and Vegetable 112
Pneumonitis, Cerebro-Spinal 134
Penis, Phagadena 138
Pregnancy, Extra-Uterine 152
Poisoning by Chloral and Morphia, etc. 180, 291
Pariera Breria. By Squibb 190
Patella, Fracture of 199
Pulse, Photographing of 207
Peritonitis, Acute 321
Placental Respiration, Artificial 369

**R.**

Ribs, Fracture of, Collodion in 63
Rat Bite, Cures of 3, 51
Rheumatism, Syphilitic and Intestinal 24, 70, 222, 267
Rush Medical College 25
Rectum, Stricture of 40
Round Ligament, Hydrocele of 74
Respiratory Murmurs 75, 221

**S.**

Skin, Diseases of 1
Small-Pox, Epidemics, etc. 223, 141, 78, 76, 9, 12, 23, 30, 40
Subscribers in City 10
Surgery, Principles and Practice of 10
Students in Vienna 14
Sargent, E. H., Druggist 25
Scarlatina 31
Skull, Fracture of 36
Splenitis and Ague 38
Syphilis, Diagnosis by Microscope 57, 72, 103, 135, 229

**T.**

Tobacco-Neurosis 357
Teucrium Marum in Aural Polypus 225
Tibia, Reproduction of 140
Tænia Solium in Child Five Days Old 13
Tuberculosis 37
Tonsils, Excision of 40
Tetanus, Chloral Hydrate in 62, 64, 91, 267
Taylor, Charles Bell 76
Transactions of Medical Societies 80
Toner, Lectures 105
Taxis in Strangulated Hernia 124
Translations, Original 374

**U.**

Urethra, Occlusion of 33, 263
Uterus, Inversion of 36, 113
Uterus, Rupture of 151
Uterus, Excision of 266
Utero-Placental Adhesion 353

**V.**

Varix, Concealed 228
Vertebræ, Caries of 178
Venesection 144
Venereal Ulcers, Treatment of 140, 144, 190
Vaccination—Papers 47, 64, 141
Vaccinal Syphilis 59
Vaccine Virus, Preservation of 64, 220
Vagina, Absence of 93
Variola, Scarlatina, Measles, in Same Patient 101, 152

**W.**

Whooping Cough and Cod Liver Oil 64, 171
Women, Diseases of 80
Wasp-Stings 104
Wounds, New Dressing 136
West Virginia State Medical Society 186
Washington Territory, Climate of 209
Wisconsin State Medical Society 249

| | |
|---|---|
| Measles | 1 |
| Meningitis | 9 |
| " cerebro-spinal | 3 |
| " tubercular | 2 |
| Metritis, puerperal | 1 |
| Metro peritonitis | 2 |
| Nephritis hepatitis | 1 |
| Old age | 9 |
| Paralysis | 1 |
| Peritonitis | 2 |
| " puerperal | 2 |
| Pneumonia | 31 |
| " typhoid | 3 |
| Potts' disease | 1 |
| Pyæmia | 1 |
| " and erysipelas | 1 |
| Rheumatism | 2 |
| Scrofula | 2 |
| Small-pox | 47 |
| Spine, injury of | 1 |
| Syphilis | 1 |
| Tabes mesenterica | 15 |
| Suicide by cutting throat | 1 |
| " " hanging | 1 |
| " " shooting | 1 |
| Throat, malignant sore | 1 |
| Uterus, hæmorrhage of | 2 |
| Uremic poisoning | 1 |
| Whooping cough | 4 |
| Total | 526 |
| Premature births | 6 |
| Still births | 47 |
| Total | 53 |

COMPARISON.

| | |
|---|---|
| Deaths in December, 1871 | 529 |
| " " preceding month in 1871 | 516 |
| Increase | 13 |

AGES.

| | |
|---|---|
| Under one year | 134 |
| One year to two | 32 |
| Two years to three | 36 |
| Three years to four | 11 |
| Four years to five | 18 |
| Five years to ten | 35 |
| Ten years to twenty | 29 |
| Twenty years to thirty | 62 |
| Thirty years to forty | 62 |
| Forty years to fifty | 48 |
| Fifty years to sixty | 17 |
| Sixty years to seventy | 27 |
| Seventy years to eighty | 13 |
| Eighty years to ninety | 5 |
| Total | 529 |
| Males | 284 |
| Females | 245 |
| Total | 529 |
| Married | 138 |
| Single | 241 |
| Total | 529 |
| Colored | 8 |
| White | 521 |
| Total | 529 |

NATIVITES.

| | |
|---|---|
| Austria | 2 |
| Bohemia | 7 |
| Canada | 4 |
| Native Chicago | 61 |
| Foreign " | 172 |
| New York and other parts | 78 |
| Denmark | 5 |
| England | 18 |
| Germany | 89 |
| Holland | 2 |
| Ireland | 52 |
| Isle of Man | 1 |
| Norway | 12 |
| Poland | 1 |
| Scotland | 6 |
| Shetland Islands | 1 |
| Sweden | 11 |
| Switzerland | 2 |
| Unknown | 5 |
| Total | 529 |
| Deaths daily per month | 17 |
| Rain fell during month (inches) | 3.440 |

MORTALITY BY WARDS, DECEMBER, 1871.

| Wards. | No. Deaths. | Pop'n (1871). | Percentage. |
|---|---|---|---|
| 1 | — | 8,103 | one death in —— |
| 2 | 2 | 13,449 | " " " 13,449 |
| 3 | 33 | 17,934 | " " " 543 |
| 4 | 11 | 14,022 | " " " 1,275 |
| 5 | 15 | 14,991 | " " " 999 |
| 6 | 37 | 22,918 | " " " 619 |
| 7 | 48 | 15,590 | " " " 325 |
| 8 | 54 | 25,420 | " " " 471 |
| 9 | 49 | 30,778 | " " " 628 |
| 10 | 14 | 17,292 | " " " 1,235 |
| 11 | 21 | 19,212 | " " " 772 |
| 12 | 17 | 15,018 | " " " 883 |
| 13 | 14 | 9,740 | " " " 696 |
| 14 | 12 | 9,339 | " " " 779 |
| 15 | 77 | 25,706 | " " " 334 |
| 16 | 15 | 16,380 | " " " 1,092 |
| 17 | 13 | 18,814 | " " " 1,449 |
| 18 | 12 | 18,805 | " " " 1,567 |
| 19 | — | 9,237 | " " " —— |
| 20 | 1 | 14,522 | " " " 14,522 |
| Accidents | 23 | —— | —— |
| Barracks | 10 | —— | —— |
| County Hosp. | 13 | —— | —— |
| Foundling Home | 2 | —— | —— |
| Home for Friendless | 2 | —— | —— |
| Hosp. W'm'n & Chil. | 1 | —— | —— |
| Manslaughter | 1 | —— | —— |
| Police Station | 1 | —— | —— |
| Mercy Hospital | 12 | —— | —— |
| St. Jo. Orph. Asylum | 1 | —— | —— |
| St. Luke's Hospital | 5 | —— | —— |
| Scammon " | 2 | —— | —— |
| Small-pox " | 8 | —— | —— |
| Suicides | 3 | —— | —— |
| Washington'n Home | 1 | —— | —— |
| Total | 529 | | |

MONEY RECEIPTS, DECEMBER 15TH, 1871, TO JANUARY 8TH, 1872.—Dr.'s J. B. Le Bland, $2.00; D. B. lor, 10.00; O. W. Sadler, 6.00; S. A McWilliams, 3.00; T. N. Livesay, 3.00; J. W. Jones, 3.00; C. N. Cooper, 3.00; A. D. Andrews, 3.00; W. T. Didlake, 6.00; O. T. Bundy, 3.00; Dr. Bell, 3.00; J. J. Rousseau, 3.00; W. M. Lander, 3.00; J. B. Cloud, 9.00; M. Risch, 3.00; C. J. Adams, 3.00; D. B. Copp, 3.00; M. M. Davis, 2.00; E. Winchester, 12.00; D. Duchett, 4.00; G. W. Dodge, 3.00; J. E. Best, 3.00; L. H. Kennedy, 6.00; J. H. Breeden, 3.00.

